# Insects and the Dissemination of Infectious Diseases

**Published:** 1899-09-16

**Authors:** 


### INSECTS AND THE DISSEMINATION OF INFECTIOUS DISEASES.

Several papers have lately appeared dealing with the part played by insects in the spread of disease, and it would now seem to be proved to demonstration that instead of being an exceptional and comparatively rare mode of distribution the portage of infection by flies and other winged carriers of disease is one of the commonest of events. In a paper read at Portsmouth, Dr. George H. F. Nuttall, of Cambridge, showed that the part played by insects in the spread of bacterial disease might be active or passive, but more especially the latter. As examples of passive carriers of disease, he instanced common flies, which, although incapable of "biting," might from the nature of the food they sought carry pathogenic bacteria about on their bodies or within their alimentary tracts, and might deposit them on lesions of the mucous membrane or skin, or on food. In this way anthrax, plague, cholera, typhoid fever, and other diseases might be transported from place to place. In regard to anthrax this had been recognised for years past. The presence of many dead flies during plague epidemics had been recorded in some of the older chronicles; and in 1894 Yersin not only found many dead flies lying about his laboratory, where animals which had died of plague were being examined, but in one case proved by inoculation that the fly he examined contained the plague bacilli. In 1897 Dr. Nuttall made experiments with flies which were fed with the organs of animals dead of plague, and it was found that such flies still contained virulent plague bacilli in their fæces 48

hours and longer after they had last been fed upon plague organs. It was thus proved that flies could live a considerable time whilst carrying plague bacilli in a virulent state. About the same time Hankin made similar observations in regard to certain ants which had been fed upon rats dead of plague. The possibility of the infection of cholera being carried by flies was pointed out by Nicholas in 1873, long before the discovery of the microbic nature of this infection, and the fact has been practically proven in several more recent epidemics. As to typhoid fever, Dr. Nuttall says that it seems almost certain that flies may infect themselves by feeding on typhoid excreta and then transport the germs to food which is left exposed. Among the other diseases in which flies play this passive part may be mentioned the case of Egyptian ophthalmia, which is almost certainly carried from eye to eye by their means. As to the active part ascribed by some writers to blood-sucking flies in the propagation of certain bacterial diseases Dr. Nuttall is very sceptical, and he says that although certain clinical observers have reported cases arising in such a manner, experimental proof is wanting.

Experiments made by him on animals with plague, anthrax, &c., in which insects were allowed to bite animals dying of these diseases and were then immediately afterwards transferred to healthy animals, all gave negative results, not a single case of infection occurring. At the same time it is clear that a bug or a flea filled with the blood from a patient containing plague bacilli may serve as a passive carrier, and if such an insect were crushed, and the skin scratched by nails soiled with the blood it contained, infection might readily occur. Insects may also serve as carriers of diseases such as malaria and many others due to animal parasites, and in so doing may operate either actively or passively, and also while acting as intermediate hosts or without so doing.

In regard to the more clinical aspect of this subject of the insect portage of infection many discussions have taken place within the last few months in America, where a strong feeling has arisen to the effect that the *rôle* played by flies in the dissemination of typhoid fever among the troops assembled for the war in Cuba and in the Phillipines was a most important one. In a discussion at the Buffalo Sanatory Club a few months ago, which was joined in by many surgeons who had been present in the camps, a very strong opinion was expressed as to the part played by flies in distributing the infection, and as to the importance of prompt covering of the excreta and the immediate application of such forms of disinfectants as would prevent the distribution of the infection by flies.

In regard to the same subject reference may also be made to various recent reports of medical officers of health in England showing the greater prevalence of typhoid fever in midden towns than in those provided with water-closets, notwithstanding that the water supply might be above suspicion, a prevalence which is probably to be explained by the facility with which flies carry the infection from the middens or tubs to the food within the neighbouring dwellings.

The influence that the animals which live along with man exercise upon his health is becoming every day more apparent. Not only may the stranger within our gates be the bearer of infection, but also our cattle, our pigs, our fowls, our rabbits, our flies, our fleas, our bugs, and all the parasites which grow on and around mankind; all may in one way and another become carriers of infection, and may thus spread disease.

